# Effect of lipid-lowering medications on oxidized phospholipids in individuals with elevated LIPOPROTEIN(a)

**DOI:** 10.1016/j.athplu.2025.09.003

**Published:** 2025-09-04

**Authors:** Amalia Despoina Koutsogianni, Fotios Barkas, Constantinos Tellis, Alexandros Tselepis, George Liamis, Sotirios Tsimikas, Evangelos Liberopoulos

**Affiliations:** a Department of Internal Medicine, Faculty of Medicine, School of Health Sciences, University of Ioannina, Ioannina, Greece; b Department of Chemistry, Division of Organic Chemistry and Biochemistry, School of Natural Sciences, University of Ioannina, Ioannina, Greece; c Atherothrombosis Research Center, Laboratory of Biochemistry, Department of Chemistry, School of Natural Sciences, University of Ioannina, Ioannina, Greece; d Division of Cardiovascular Medicine, Sulpizio Cardiovascular Center, University of California San Diego, La Jolla, CA, USA; e 1st Propedeutic Department of Medicine and Diabetes Center, School of Medicine, National and Kapodistrian University of Athens, Laiko General Hospital, Athens, Greece

**Keywords:** Lipoprotein(a), Oxidized phospholipids, Plasminogen, Statins, Ezetimibe, PCSK9 inhibitors

## Abstract

**Background/introduction:**

Oxidized phospholipids (OxPLs) are bound to apolipoprotein B-100 (OxPL-apoB) and apolipoprotein(a) [OxPL-apo(a)] and are present freely within the phospholipid shell of apoB-containing lipoproteins. OxPLs have been linked with the pro-inflammatory properties of lipoprotein(a) [Lp(a)]. OxPLs carried on plasminogen (OxPL-PLG) may extend the time to fibrinolysis.

**Purpose:**

To evaluate the effect of lipid-lowering medications on OxPLs levels in individuals with elevated Lp(a) concentrations.

**Methods:**

In this prospective study, patients (n = 70) with Lp(a) levels ≥75 nmol/L were assigned to 3 treatment regimens according to current guidelines: high-intensity statin monotherapy (n = 28), ezetimibe added to high-intensity statin (n = 31) and proprotein convertase subtilisin/kexin type 9 inhibitor (PCSK9i) added to high-intensity statin plus ezetimibe (n = 11). Follow-up duration was 3 months.

**Results:**

Patients had a mean age of 51 ± 15 years, 40 % were male, 39 % were diagnosed with familial hypercholesterolemia, 16 % had atherosclerotic cardiovascular disease, and 36 %, 33 % and 15 % were at very high, high, and moderate cardiovascular risk, respectively. Lp(a) levels did not change significantly with high-intensity statin and add-on ezetimibe but significantly decreased with add-on PCSK9i treatment. OxPL-apoB and OxPL-apo(a) significantly increased, while OxPL-PLG significantly decreased with both high-intensity statin and add-on ezetimibe. Add-on PCSK9i treatment was associated with no significant changes in OxPL-apoB, OxPL-apo(a) and OxPL-PLG levels.

**Conclusions:**

Among patients with elevated Lp(a), both high-intensity statin and add-on ezetimibe significantly increased OxPL-apoB and OxPL-apo(a) levels, while significantly decreased OxPL-PLG levels. Add-on PCSK9i had no significant effect on OxPLs levels. The clinical implications of these findings should be further explored.

## Highlights

• Prospective study including patients with Lp(a) ≥75 nmol/L and dyslipidemia.
• In naïve patients receiving high-intensity statins, OxPL-apoB and OxPL-apo(a) levels increased, while OxPL-PLG decreased.
• The same observations were noted in patients receiving ezetimibe as add-on to high-intensity statins.
• In patients receiving add-on PCSK9i to high-intensity statin plus ezetimibe, a significant decline was noted in Lp(a).
• In patients receiving add-on PCSK9i to high-intensity statin plus ezetimibe, no significant changes were noted in any OxPL.

## Graphical abstract

A prospective study including 70 adult patients with Lp(a) levels ≥75 nmol/L and dyslipidemia. Patients were assigned to 3 treatment regimens according to current guidelines: high-intensity statin monotherapy (n = 28), ezetimibe added to high-intensity statin (n = 31) and proprotein convertase subtilisin/kexin type 9 inhibitor (PCSK9i) added to high-intensity statin plus ezetimibe (n = 11). Lipoprotein(a) [Lp(a)], oxidized phospholipids (OxPLs) associated with apolipoprotein B-100 (OxPL-apoB), OxPLs associated with apolipoprotein(a) [OxPL-apo(a)], plasminogen (PLG) and OxPLs associated with PLG (OxPL-PLG) were measured at baseline and 3 months after the intervention. Lp(a) levels did not significantly change with high-intensity statin and add-on ezetimibe but significantly decreased with PCSK9i treatment. OxPL-apoB and OxPL-apo(a) significantly increased, while OxPL-PLG significantly decreased with both high-intensity statin and add-on ezetimibe. PCSK9i treatment was associated with no significant change on OxPL-apoB, OxPL-apo(a) and OxPL-PLG levels.

**Figure:**
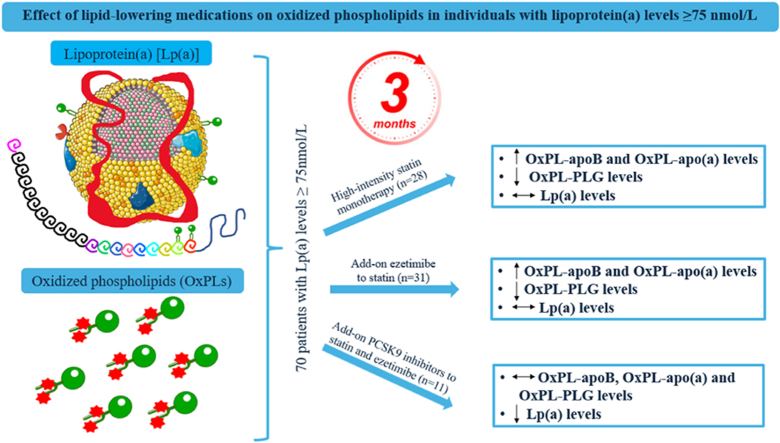

## 1 Introduction

Atherosclerotic cardiovascular disease (ASCVD) remains the leading cause of mortality worldwide despite intensive lipid-lowering treatment [1]. Oxidized phospholipids (OxPLs) contribute to atherosclerosis by promoting inflammation and oxidative modification of lipoproteins, while plasminogen (PLG) plays a central role in fibrinolysis that can be modified by OxPLs, potentially affecting thrombotic risk [2].

Lipoprotein(a) [Lp(a)], structurally similar to low-density lipoprotein (LDL), is strongly linked to ASCVD [2]. Apolipoprotein(a) [apo(a)] shares homology with PLG, potentially interfering with fibrinolysis [2]. Lp(a) is the preferential lipoprotein carrier of OxPLs in the bloodstream [2].

OxPLs, arising from oxidative modifications of phospholipids, are involved in inflammation, immunity, and lipid metabolism [2,3]. They primarily associate with apolipoprotein(a) [OxPL-apo(a)] and apolipoprotein B-100 (OxPL-apoB), key component of various lipoproteins, including Lp(a) [[4], [5], [6]]. OxPLs accumulate in atherosclerotic lesions and impact vascular endothelial cells, immune cells, and coagulation, influencing the pathogenesis of atherosclerosis and thrombosis [2,3]. Increased levels of OxPL-apoB and OxPL-apo(a) are associated with higher risks of cardiovascular events [7,8].

PLG is the precursor to plasmin, responsible for fibrinolysis [9]. PLG exhibits pleiotropic functions in extracellular matrix remodeling, cell migration, and inflammatory signaling [9]. OxPLs are also bound covalently to PLG, and when they are enzymatically removed are associated with longer time to fibrinolysis [10]. Increased levels of OxPL-PLG are associated with lower risk of acute thrombotic events [9,11].

The effects of currently used lipid-lowering medications on OxPLs levels remain unclear. Statins show mixed effects on OxPL-apoB [12,13], with limited data on OxPL-apo(a) [14]. Ezetimibe's effect on OxPL-apoB and OxPL-apo(a) is not well studied. Proprotein convertase subtilisin/kexin type 9 inhibitors (PCSK9i) have a neutral effect on OxPL-apoB [15,16], but data for any effect on OxPL-apo(a) are lacking. Novel Lp(a)-lowering drugs (pelacarsen, olpasiran, muvalaplin) have shown significant reductions in OxPL-apoB and OxPL-apo(a) levels [[17], [18], [19]].

In this study, we aimed to evaluate the impact of high-intensity statins, add-on ezetimibe and add-on PCSK9i on OxPL-apoB, OxPL-apo(a) and OxPL-PLG levels in individuals with elevated Lp(a) concentrations.

## 2 Materials and methods

### 2.1 Study population

**Fig. 1:**
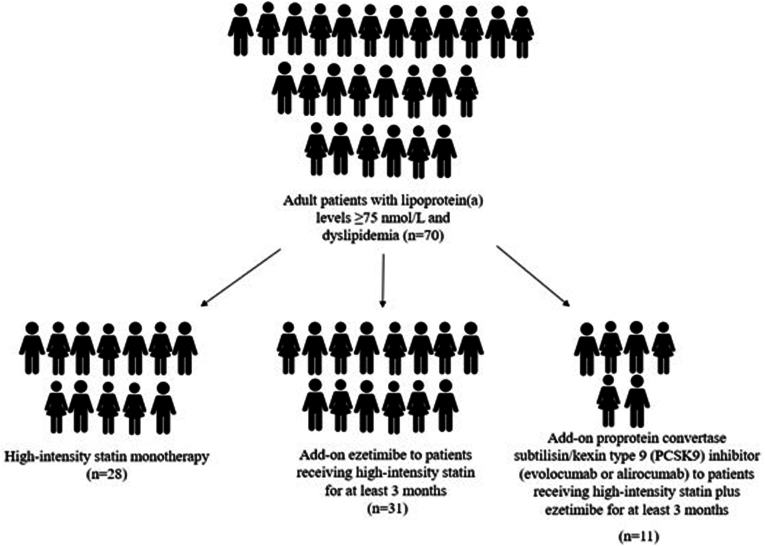
Study design.

This was a prospective study including consecutive adult patients with elevated Lp(a) levels (≥75 nmol/L according to the Hospital Biochemical Laboratory) attending the Outpatient Lipid Clinic of the University Hospital of Ioannina. Patients were non-randomly allocated to 3 treatment groups according to the national guidelines for the management of dyslipidemias [20]. All study participants were of Caucasian origin. The 10-year total cardiovascular risk was estimated with the Systematic Coronary Risk Evaluation (SCORE) 2 model for apparently healthy subjects aged 40–69 years and with the SCORE 2 old persons (OP) model in those aged ≥70 years [21]. We included a group of naïve patients who received high-intensity statin monotherapy, a group of patients on stable high-intensity statin treatment who received add-on ezetimibe and a group of patients on stable high-intensity statin treatment plus ezetimibe who received add-on PCSK9i (evolocumab or alirocumab) (Fig. 1). The patients were on high-intensity statin treatment and high-intensity statin treatment plus ezetimibe for at least 3 months before adding ezetimibe or a PCSK9i, respectively. High-intensity statin treatment included rosuvastatin 20–40 mg or atorvastatin 40–80 mg [20]. The follow-up duration was 3 months.

The study protocol conforms to the ethical guidelines of the 1975 Declaration of Helsinki, was approved by the Ethics Committee of the University General Hospital of Ioannina, Greece (896/21-12-2020) and written informed consent was obtained from each patient.

### 2.2 Clinical and laboratory assessments

A complete assessment of clinical and laboratory profile was performed at baseline visit and 3 months after the initiation/modification of lipid-lowering treatment. Demographic and clinical characteristics included: i) sex, ii) age, iii) smoking history, iv) concomitant diseases with a particular emphasis on established ASCVD (coronary artery disease, stroke, peripheral artery disease and carotid stenosis ≥50 %), and cardiometabolic risk factors, v) calcific aortic valve stenosis (CAVS), vi) body mass index (BMI), and vii) blood pressure (BP). Heterozygous familial hypercholesterolemia (HeFH) was defined according to the Dutch Lipid Clinic Network (DLCN) criteria [22]. Venous blood samples were obtained both into sterile Vacutainer-SST II advance tubes (Becton-Dickinson, Plymouth, UK) and into a vacutainer containing potassium EDTA, in the morning after 12 h fasting. Serum and plasma samples were stored at −80 °C. Serum concentrations of total cholesterol (TC), and triglycerides (TG) were determined enzymatically on the Olympus AU600 Clinical Chemistry Analyser (Olympus Diagnostica, Hamburg, Germany). High-density lipoprotein cholesterol (HDL-C) was determined by a direct assay (Olympus Diagnostica, Hamburg, Germany). Low-density lipoprotein cholesterol (LDL-C) was calculated using the Friedewald formula if TG levels were <400 mg/dL (4.5 mmol/L).

### 2.3 Determination of OxPL-apoB, OxPL-apo(a), OxPL-PLG

OxPL-apoB and OxPL-apo(a) reflect the amount of OxPLs present on a normalized similar amount of apoB-100-containing lipoproteins or Lp(a) captured on the microtiter well plates from each patient, irrespective of the total plasma concentration of each [23]. These assays do not measure total OxPLs present on all apoB-100-containing or Lp(a) particles, but the relative enrichment of OxPLs per lipoprotein particle, and therefore can be considered as reflecting the carrying capacity of OxPLs per apoB-100-containing or Lp(a) particle [23]. Although Lp(a) is the major lipoprotein carrier of OxPLs [2], measuring OxPL-apoB provides additional information on the OxPL content in other apoB-100 containing particles, such as LDL and VLDL, thereby offering a broader view of OxPL distribution and treatment effects beyond Lp(a) alone. For the OxPL-apoB assay, all apoB-100 particles [very low-density lipoprotein, intermediate-density lipoprotein, LDL, Lp(a)] are captured with similar affinity on microtiter well plates with monoclonal antibody MB47 and in proportion to their plasma concentration [23]. For OxPL-apo(a), the plates only contain Lp(a) captured with monoclonal antibody LPA4 [23]. For this reason, OxPL-apo(a) levels tend to be higher than OxPL-apoB levels, as the microtiter well plates are more heavily concentrated with the main lipoprotein carrier of OxPL [23].

Briefly, plasma OxPL-apoB, OxPL-apo(a) and OxPL-PLG levels were quantified with a chemiluminescent ELISA using the murine monoclonal E06 antibody, which specifically binds to the phosphocholine (PC) headgroup of OxPLs on apoB, apo(a)-containing particles and PLG, respectively, at the University of California at San Diego, California, USA [10,23,24]. The levels of OxPL-apoB, OxPL-apo(a) and OxPL-PLG were determined using microtiter well plates coated with a) apoB-100 specific monoclonal antibody MB47, b) specific antibody apolipoprotein(a) LPA4, c) mouse monoclonal anti-human plasminogen antibody, followed by E06 antibody respectively as previously described [10,24,25]. Units report as nmol/L of PC equivalents of PC-containing oxidized phospholipids.

### 2.4 Lp(a) plasma levels

Lp(a) was measured using a validated, isoform independent enzyme-linked immunosorbent assay (ELISA) traced to WHO/IFCC reference material SRM 2B as previously described, which ensures accurate measurement of Lp(a) levels irrespective of the diverse isoform sizes, overcoming limitations of earlier techniques [26]. The assay is based on the use of LPA4 monoclonal antibody, to capture plasma Lp(a), and a second LPA-KIV9 monoclonal antibody against a unique antigenic site on KIV9. The range of the LPA4/LPA-KIV9 ELISA is 0.27–1.402 nmol/L and the method met strict criteria for accuracy, linearity, recovery, and precision.

### 2.5 Statistical analysis

Continuous variables were tested for normality by the Kolmogorov-Smirnov test. Data are presented as mean ± standard deviation (SD) and median [interquartile range (IQR)] for parametric and non-parametric data, respectively. For categorical values, frequency counts and percentages were applied. Chi-square (χ^2^) test was performed for interactions between categorical values. The independent sample *t*-test (parametric and non-parametric) was used for the comparison of continuous numeric values between 2 groups. One-way analysis of variance (one-way ANOVA) was performed to assess the difference of the variables of interest between ≥2 groups. Multivariate analysis of covariance (MANCOVA) was used for the comparison of continuous numeric values between ≥2 groups, adjusting for the baseline value of the variable of interest as well as for key covariates including age, sex, LDL-C, Lp(a), and ASCVD status. Correlation analyses between Lp(a), LDL-C, OxPL-apoB, and OxPL-apo(a) at baseline and follow-up as well as between the changes observed over time were performed using Pearson's correlation coefficient. Analyses were performed for the entire cohort as well as each treatment group. Two-tailed significance was defined as *p* < 0.05. Analyses were performed with the SPSS v21.0 software (SPSS Statistics for Windows, Version 28.0. Armonk, New York, NY, USA: IBM Corp).

## 3 Results

### 3.1 Baseline characteristics

In total, 70 subjects were included. Specifically, 28 naïve patients received high-intensity statin monotherapy, 31 patients on stable high-intensity statin treatment received add-on ezetimibe and 11 patients on stable high-intensity statin treatment plus ezetimibe received add-on a PCSK9i. In the statin monotherapy group, 100 % of patients received rosuvastatin (median dose 20 mg). In the statin plus ezetimibe group, 77 % were receiving rosuvastatin (median dose 20 mg) and 23 % atorvastatin (median dose 40 mg). In the PCSK9i group, 55 % were receiving rosuvastatin (median dose 40 mg) and 45 % atorvastatin (median dose 40 mg). All therapeutic interventions were well tolerated, and no adverse events were reported during the study. Baseline characteristics are presented in Table 1. Mean age was 51 ± 15 years, 40 % were male, 39 % were diagnosed with HeFH, 16 % had ASCVD, and 36 %, 33 % and 15 % were at very high, high, and moderate cardiovascular risk, respectively. Patients who received add-on ezetimibe ± PCSK9i had significantly increased prevalence of arterial hypertension and type 2 diabetes compared with patients on high-intensity statin monotherapy (*p* = 0.026 and *p* = 0.033, respectively). In addition, patients on triple therapy had significantly increased prevalence of HeFH, ASCVD and chronic kidney disease compared with the other 2 groups (*p* = 0.000, *p* = 0.000 and *p* = 0.021, respectively, for the comparison with the high-intensity statin monotherapy group and *p* = 0.000, *p* = 0.000 and *p* = 0.015, respectively, for the comparison with the add-on ezetimibe group).

**Table 1:** Study participant baseline characteristics.

	High-intensity statin monotherapy	Add-on ezetimibe to statin	Add-on PCSK9i to statin and ezetimibe	Total sample
**Number of patients (N)**	28	31	11	70
**Gender**				
Male	32 %	42 %	55 %	40 %
**Age (years)**	45 (±16)	54 (±13)	55 (±14)	51 (±15)
**Smoking**				
Never smoker Former smoker Current smoker	64 % 11 % 25 %	58 % 16 % 26 %	60 % 30 % 10 %	61 % 16 % 23 %
**Cardiovascular Risk Categories**				
Low risk	26 %	11 %	9 %	17 %
Intermediate risk	22 %	15 %	0	15 %
High risk	39 %	37 %	9 %	33 %
Very high risk	13 %	37 %	82 %	36 %
**Arterial hypertension**	11 %	36 %**∗**	36 %	26 %
**Type 2 diabetes**	4 %	23 %**∗**	20 %	15 %
**Heterozygous familial hypercholesterolemia**	21 %	32 %	100 %**∗^#^**	39 %
**Coronary heart disease**	0	7 %	64 %**∗^#^**	13 %
**Stroke**	0	3 %	9 %	3 %
**Carotid stenosis**	4 %	0	9 %	3 %
**Peripheral artery disease**	0	0	18 %**∗^#^**	3 %
**Atheroscleroticcardiovascular disease**	4 %	10 %	100 %**∗^#^**	16 %
**Calcific aortic valve stenosis**	0	0	9 %	1 %
**Chronic kidney disease**	0	0	18 %**∗^#^**	3 %

Data are presented as N (%). Parametric variables are presented as mean ± SD and non-parametric as median (IQR).

∗*p* < 0.05 for the comparison with high-intensity statin monotherapy.

#*p* < 0.05 for comparison with high-intensity statin plus ezetimibe.

Abbreviations: LDL-C; Low-density lipoprotein cholesterol, PCSK9i; proprotein convertase subtilisin/kexin type 9 inhibitors.

### 3.2 Changes in lipids

Table 2 depicts lipid levels at baseline, as well as the effect of lipid-lowering medications. All interventions significantly reduced TC, LDL-C and apoB, with PCSK9i demonstrating the largest reductions. No significant change of Lp(a) levels was observed with high-intensity statin and add-on ezetimibe (*p* = 0.24 and *p* = 0.06, respectively). Add-on PCSK9i significantly decreased Lp(a) levels by 43.1 % (*p* < 0.05).

**Table 2:** Effect of lipid-lowering treatment on lipids, oxidized phospholipids, and plasminogen levels.

	Baseline Visit	After 3 Months	% Change within each group
**TC, mg/dL, mean (SD)**			
High-intensity statin (n = 28)	256 (±38)	177 (±35)	−31 % ^ǂ^
Add-on ezetimibe to statin (n = 31)	176 (±23) **∗**	151 (±23) **∗**	−14 % ^ǂ^
Add-on PCSK9i to statin and ezetimibe (n = 11)	217 (±52) **∗^#^**	138 (±43) ∗^#^	−36 % ^ǂ^
			
**TGs, mg/dL, median (IQR)**			
High-intensity statin (n = 28)	97 (57; 263)	78 (45; 371)	−20 % ^ǂ^
Add-on ezetimibe to statin (n = 31)	105 (44; 198)	81 (45; 254)	−23 %
Add-on PCSK9i to statin and ezetimibe (n = 11)	96 (72; 270)	79 (44; 363)	−18 %

**HDL-C, mg/dL, mean (SD)**			
High-intensity statin (n = 28)	60 (±15)	58 (±13)	−3 % ^ǂ^
Add-on ezetimibe to statin (n = 31)	54 (±16)	54 (±14)	0
Add-on PCSK9i to statin and ezetimibe (n = 11)	52 (±12)	51 (±15)	−2 %
			
**LDL-C, mg/dL, mean (SD)**			
High-intensity Statin (n = 28)	174 (±34)	99 (±27)	−43 % ^ǂ^
Add-on ezetimibe to statin (n = 31)	101 (±19) **∗**	79 (±15) **∗**	−22 % ^ǂ^
Add-on PCSK9i to statin and ezetimibe (n = 11)	140 (±45) **∗^#^**	63 (±30) ∗**^#^**	−55 % ^ǂ^

**Lp(a), nmol/L, median (IQR)**			
High-intensity Statin (n = 28)	131.4 (100.4; 171.9)	138.7 (111.0; 195.5)	+5.5 %
Add-on ezetimibe to statin (n = 31)	128.5 (75.0; 184.8)	148.2 (106.6; 179.9)	+15.3 %
Add-on PCSK9i to statin and ezetimibe (n = 11)	139.6 (75.0; 278.9)	84.8 (39.5; 210.9)	−43.1 % ^ǂ^

**ApoB, mg/dL, mean (SD)**			
High-intensity statin (n = 28)	102 (±27)	70 (±18)	−31 % ^ǂ^
Add-on ezetimibe to statin (n = 31)	85 (±26) ∗	65 (±13)	−24 % ^ǂ^
Add-on PCSK9i to statin and ezetimibe (n = 11)	107 (±22)^#^	59 (±19)	−45 % ^ǂ^
			
**OxPL-apoB, nmol/L, median (IQR)**			
High-intensity statin (n = 28)	27.9 (19.7; 33.0)	28.7 (21.2; 49.2)	+2.9 % ^ǂ^
Add-on ezetimibe to statin (n = 31)	27.5 (21.3; 37.8)	35.4 (19.8; 57.9)	+28.7 % ^ǂ^
Add-on PCSK9i to statin and ezetimibe (n = 11)	24.8 (16.7; 45.4)	21.7 (5.7; 55.3)	−12.5 %
			
**OxPL-apo(a), nmol/L, median (IQR)**			
High-intensity statin (n = 28)	11.9 (9.7; 15.6)	23.9 (13.4; 33.1)	+100.8 % ^ǂ^
Add-on ezetimibe to statin (n = 31)	14.2 (11.6; 20.0)	19.9 (13.8; 34.5)	+40.1 % ^ǂ^
Add-on PCSK9i to statin and ezetimibe (n = 11)	15.0 (9.4; 20.1)	16.8 (10.5; 39.8)	+12.0 %
			
**OxPL-PLG, nmol/L, median (IQR)**^§^			
High-intensity statin (n = 28)	257.9 (224.4; 343.2)	252.2 (175.5; 294.6)	−2.2 % ^ǂ^
Add-on ezetimibe to statin (n = 31)	303.3 (237.9; 373.8)	246.4 (202.3; 333.3)	−18.8 % ^ǂ^
Add-on PCSK9i to statin and ezetimibe (n = 11)	283.6 (170.4; 380.2)	268.8 (194.9; 299.6)	−5.2 %
			
**Total Plasminogen, mg/dL, median (IQR)**			
High-intensity statin (n = 28)	10.1 (7.5; 12.6)	10.3 (7.9; 13.3)	+2.0 %
Add-on ezetimibe to statin (n = 31)	10.7 (9.1; 14.0)	11.2 (7.6; 12.5)	+4.7 %
Add-on PCSK9i to statin and ezetimibe (n = 11)	11.3 (8.5; 14.3)	12.0 (9.7; 13.6)	+6.2 %

ǂ *p* < 0.05 for the comparison within each group.

∗*p* < 0.05 for the comparison with patients treated with high-intensity statin.

#*p* < 0.05 for the comparison with patients treated with high-intensity statin plus ezetimibe.

§p values were calculated after excluding 4 outliers.

Abbreviations: TC; Total Cholesterol, TGs; Triglycerides, HDL-C; High-density Lipoprotein Cholesterol, LDL-C; Low-density Lipoprotein Cholesterol, Lp(a); lipoprotein(a), ApoB; apolipoprotein B-100, PCSK9i; proprotein convertase subtilisin/kexin type 9 inhibitors, OxPL-apoB; oxidized phospholipids on apolipoprotein B-100, OxPL-apo(a); oxidized phospholipids on apolipoprotein(a), OxPL-PLG; oxidized phospholipids on plasminogen.

### 3.3 Changes in OxPL-apoB and OxPL-apo(a)

Table 2 depicts OxPL-apoB and OxPL-apo(a) levels at baseline as well as the effect of lipid-lowering medications.

**Fig. 2:**
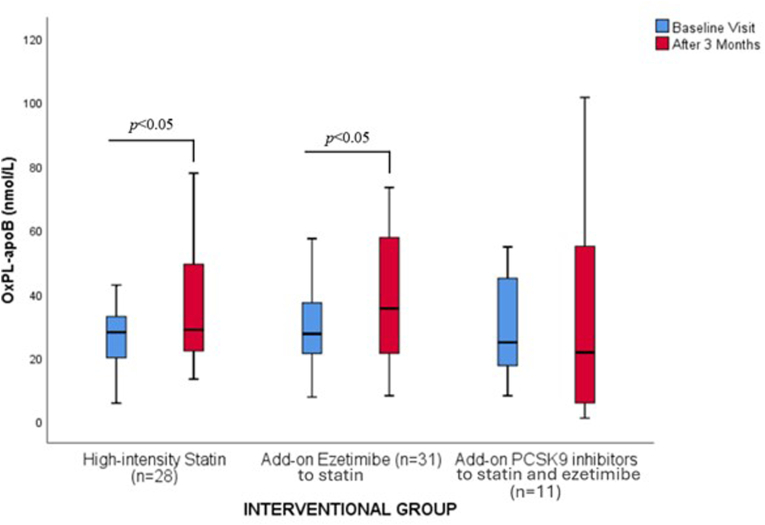
Effect of lipid-lowering treatment on the levels [median (IQR)] of oxidized phospholipids on apolipoprotein B-100 (OxPL-apoB).

**Fig. 3:**
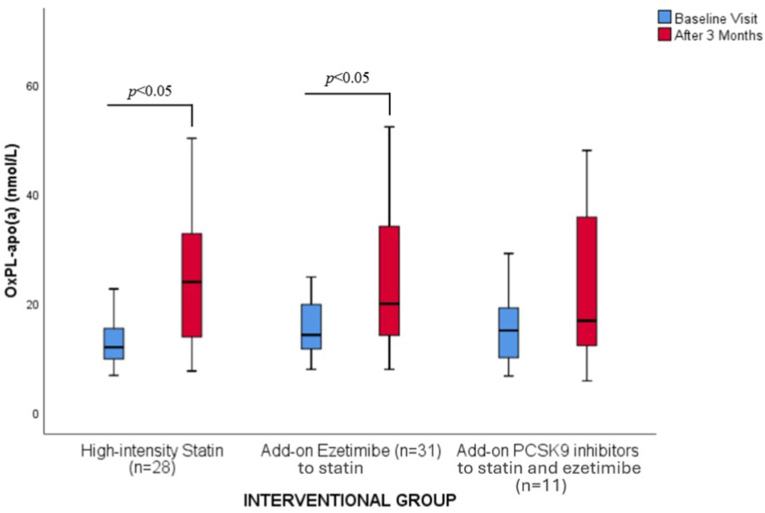
Effect of lipid-lowering treatment on the levels [median (IQR)] of oxidized phospholipids on apolipoprotein(a) levels [OxPL-apo(a)].

Both high-intensity statins and add-on ezetimibe significantly increased OxPL-apoB by 2.9 % and 28.7 % (Table 2 and Fig. 2), respectively, as well as OxPL-apo(a) by 100.8 % and 40.1 % (Table 2 and Fig. 3), respectively. Add-on PCSK9i resulted in a numerical decrease of OxPL-apoB by 12.5 % (*p* = NS) and a numerical increase of OxPL-apo(a) by 12 % (*p* = NS) (Table 2 and Fig. 2, Fig. 3).

**Fig. 4:**
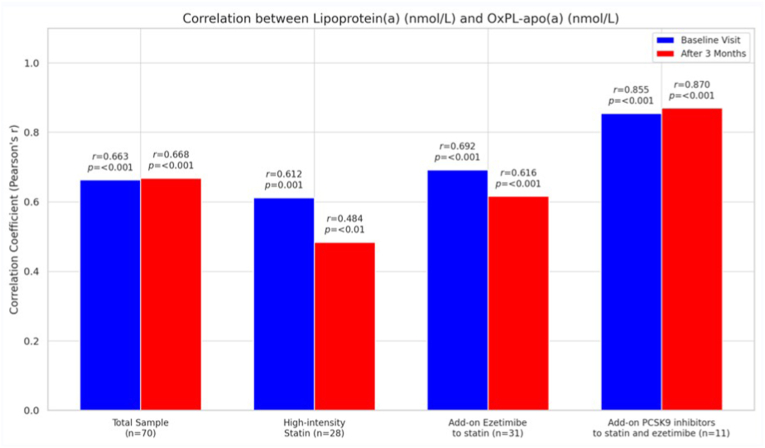
Correlation between lipoprotein(a) and oxidized phospholipids on apolipoprotein(a) [OxPL-apo(a)] levels at baseline and after 3 months of lipid-lowering treatment.

In the total population, correlation analyses at baseline revealed a significant association between Lp(a) and OxPL-apo(a) (*r* = 0.663, *p* < 0.001, Fig. 4) as well as between Lp(a) and OxPL-apoB (*r* = 0.771, *p* < 0.001), while LDL-C was not significantly correlated with OxPL-apoB (*r* = −0.190, *p* = 0.124). Similar patterns were observed at 3 months, with Lp(a) showing significant correlations with both OxPL-apo(a) (*r* = 0.668, *p* < 0.001, Fig. 4) and OxPL-apoB (*r* = 0.802, *p* < 0.001), and LDL-C not significantly associated with OxPL-apoB (*r* = −0.020, *p* = 0.870). Correlation analyses based on changes from baseline to follow-up showed no significant associations between changes in OxPL-apoB and LDL-C (*r* = −0.099, *p* = 0.431) or Lp(a) (*r* = 0.147, *p* = 0.225), whereas a significant correlation was observed between changes in OxPL-apo(a) and Lp(a) (*r* = 0.332, *p* = 0.005). When analyzed by treatment group, similar correlation patterns were observed (Fig. 4).

### 3.4 Changes in PLG and OxPL-PLG

Table 2 depicts PLG and OxPL-PLG levels at baseline as well as the effect of lipid-lowering medications.

**Fig. 5:**
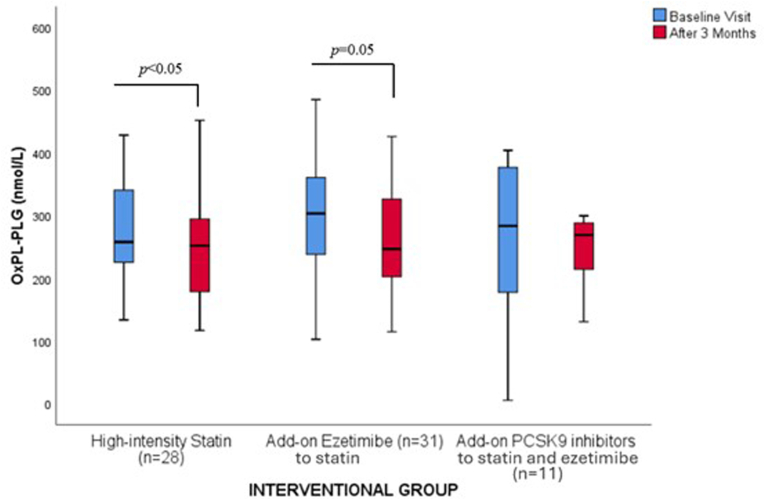
Effect of lipid-lowering treatment on the levels [median (IQR)] of oxidized phospholipids on plasminogen levels (OxPL-PLG).

Both high-intensity statins and add-on ezetimibe significantly decreased OxPL-PLG by 2.2 % and 18.8 % (Table 2 and Fig. 5), respectively, whereas no significant changes in PLG were noted. Add-on PCSK9i resulted in no significant changes in OxPL-PLG and PLG (Table 2 and Fig. 5).

## 4 Discussion

This study demonstrates several relationships among Lp(a) and OxPLs in patients with Lp(a) levels ≥75 nmol/L, receiving lipid lowering therapy. First, in naïve patients receiving high-intensity statins and in patients receiving ezetimibe add-on to high-intensity statins, OxPL-apoB and OxPL-apo(a) levels increased, along with non-significant change in Lp(a). In patients treated with add-on PCSK9i to high-intensity statin plus ezetimibe, no significant changes were seen in the OxPL-apoB and OxPL-apo(a) levels, while a significant decline was noted in Lp(a). OxPL-PLG levels were reduced in naïve patients treated with high-intensity statins and in those receiving add-on ezetimibe to high-intensity statins. In contrast, no significant changes were observed in patients on add-on PCSK9i to high-intensity statins plus ezetimibe.

### 4.1 Effect of lipid lowering medications on Lp(a)

No significant change in Lp(a) levels was observed in patients treated with high-intensity statins (+5.5 %, *p* = 0.24). The effect of statin treatment on Lp(a) levels remains an area of controversy. A large meta-analysis of 6 trials including 5256 patients randomized to various statins or placebo indicated that statins increase Lp(a) by 8–24 % [27]. However, a recent meta-analysis involving over 24,448 individuals concluded that statin therapy does not result in clinically meaningful changes in Lp(a) levels [28].

A non-significant increase in median Lp(a) levels (+15.3 %, *p* = 0.06) after 3 months of add-on ezetimibe was observed in this study. Meta-analyses have shown that ezetimibe did not significantly alter Lp(a) concentration [29] or even led to a modest reduction [30].

The add-on PCSK9i treatment was associated with a significant reduction in Lp(a) levels by 43.1 %, which is even more pronounced than the reduction of 20–30 % shown in the FOURIER and the ODYSSEY OUTCOMES trials [15].

### 4.2 Effect of lipid lowering medications on OxPL-apoB and OxPL-apo(a)

High-intensity statin treatment resulted in a significant increase in both OxPL-apoB and OxPL-apo(a) levels by 2.9 % and 100.8 %, respectively. It may be hypothesized that the reduction of LDL-C levels with high-intensity statin treatment ensues a mobilization of OxPLs from the atherosclerotic plaque into circulation, where they bind to apoB-100 particles. The increase in OxPL-apoB supports this possible mobilization. The substantial increase in OxPL-apo(a), despite unchanged Lp(a) concentration suggests that Lp(a) may become more enriched in OxPL following statin treatment. The mobilization of OxPLs from the vessel wall during high-intensity statin treatment can be attributed to several factors [31]. One key factor is the reduction in LDL-C, which changes lipid dynamics and increases OxPLs' affinity to other lipoprotein particles [31]. Additionally, statins have anti-inflammatory effects, which may help stabilize atherosclerotic plaques and reduce local inflammation [31]. This reduction in inflammation can lead to the mobilization of OxPLs as the inflammatory environment shifts [31]. Also, statin treatment improves endothelial function, potentially enhancing the endothelium's ability to process and clear OxPLs [31]. Although the mobilization of OxPLs from the vessel wall is a plausible hypothesis, it remains to be conclusively demonstrated. Statins have been associated with mixed effects on OxPL-apoB levels while limited data are available on their impact on OxPL-apo(a) levels. Data from the MIRACL trial, showed a reduction of OxPL-apoB levels by 29.7 % after treatment with high-intensity atorvastatin in patients (n = 1151) with recent ACS [12]. On the contrary, atorvastatin resulted in significant increases in OxPL-apoB by 22.9 % in the VISION trial, in which 42 patients were randomized to pitavastatin 2 mg/day or atorvastatin 10 mg/day, with no effect on OxPL-apo(a) levels [32]. In a study of 3896 patients treated with various statins an increase in OxPL-apoB by 23.8 % was noted [13]. Of importance, OxPL-apoB levels increase transiently with new statin therapy, peaking at 4 months and declining to below baseline thereafter [31]. Therefore, the 3-month follow-up of this study may coincide with the early peak in OxPL-apoB levels.

The addition of ezetimibe to high-intensity statin led to a significant increase in OxPL-apoB and OxPL-apo(a) levels by 28.7 % and 40.1 %, respectively. As with high-intensity statin, the increase in OxPL-apo(a) is greater compared with the increase in Lp(a). The underlying mechanisms behind these changes may be like those hypothesized with high-intensity statin treatment. The combination of ezetimibe with simvastatin significantly increased OxPL-apoB in one study [13].

The addition of PCSK9i resulted in a non-significant decrease in OxPL-apoB and OxPL-apo(a) levels. Similarly, the ANITSCHKOW study, in which 129 patients were randomized 1:1 to monthly evolocumab 420 mg or placebo for 16 weeks, resulted in no significant difference in the mean percent change in both OxPL-apoB and OxPL-apo(a) levels [15]. The EQUATOR study, in which 224 patients were randomized to RG7652 or placebo resulted in no significant difference in the mean percent change in OxPL-apoB [16]. However, a small but significant reduction in OxPL-apo(a) levels (−10.1 ± 31.3 nmol/L in the PCSK9i group vs 30.6 ± 90.8 nmol/L in the placebo group; *p* = 0.029) was observed [16].

The ODYSSEY OUTCOMES trial provided further insights into the effect of PCSK9 inhibition on OxPL-apoB [33]. In that study, alirocumab reduced OxPL-apoB levels by a median of 15 % after 4 months of treatment compared to placebo [33]. Importantly, baseline OxPL-apoB strongly correlated with Lp(a) levels (r = 0.68, *p* < 0.001), suggesting that the majority of OxPL-apoB is carried on Lp(a) particles [33]. Elevated OxPL-apoB levels in the placebo group were predictive of major adverse cardiovascular events (MACE), especially in people with low Lp(a) [33]. However, in the alirocumab group, neither baseline OxPL-apoB nor Lp(a) predicted MACE, suggesting that PCSK9 inhibition mitigates the OxPL-apoB- and Lp(a)-mediated risk [33].

Notably, despite the substantial Lp(a) reduction with PCSK9i, OxPL-apoB and OxPL-apo(a) levels did not decrease, which would be expected if Lp(a) was the major carrier of these species in plasma, raising questions about the underlying mechanisms. It should be noted that the assays used to measure OxPL-apoB and OxPL-apo(a) are designed to reflect the OxPL content per captured apoB or apo(a) particle, rather than total plasma OxPL levels. Therefore, the significant reduction in Lp(a) particle number may not necessarily lead to reduced OxPL-apo(a) values, particularly if the remaining particles maintain high OxPL content. Another possible explanation is that the composition of Lp(a) particles may be altered by PCSK9 inhibition, leading to a change in the types of phospholipids incorporated into these particles.

Overall, our findings suggest that the initiation of a high-intensity statin or the addition of ezetimibe results in apoB-100 and Lp(a) particles that are more enriched in OxPLs, an effect that appears to be mitigated by the addition of a PCSK9i. The reasons behind the differential effect of PCSK9i on OxPLs compared to high-intensity statins and ezetimibe remain unclear. However, this may be attributed to the fact that PCSK9i treatment was added on top of high-intensity statin and ezetimibe therapy, potentially leaving limited room for further increases in OxPLs. At both baseline and after 3 months of treatment, significant positive correlations were observed between Lp(a) and both OxPL-apo(a) and OxPL-apoB across the total study population, reinforcing that Lp(a) is the primary carrier of OxPLs in plasma. In contrast, LDL-C did not show significant correlations with OxPL-apoB suggesting that OxPL enrichment is largely independent of LDL-C concentrations. Correlation analyses for changes in these parameters showed a significant positive correlation between changes in OxPL-apo(a) and Lp(a), suggesting OxPL-apo(a) alteration may be partially explained by change in Lp(a) levels.

Measuring OxPL enrichment per particle provides valuable information on the oxidative modification of individual lipoproteins, while assessing the total plasma OxPL burden would complement this by indicating the overall atherogenic and inflammatory potential. Both parameters are clinically relevant and may provide complementary insights into cardiovascular risk.

### 4.3 Effect of lipid lowering medications on PLG and OxPL-PLG

Treatment with high-intensity statins had no effect on PLG levels but significantly decreased OxPL-PLG levels. The possible underlying mechanisms remain poorly understood. Prior studies have shown that PLG can carry OxPLs and that these OxPL modifications may interfere with PLG activation and fibrinolytic efficiency [10,34]. Statin therapy has been reported to modestly enhance fibrinolytic activity, potentially through reductions in plasminogen activator inhibitor-1 (PAI-1) levels and improved endothelial function [[9], [10], [11],^35^]. Our observed reduction in OxPL-PLG levels with high-intensity statin treatment may be relevant in this context, as prior studies have shown that OxPL adducts on PLG impair PLG function and fibrinolysis, and that their removal enhances fibrinolytic capacity [[9], [10], [11],^35^]. Add-on ezetimibe showed a similar pattern, with reduced OxPL-PLG but no significant change in total PLG. Treatment with PCSK9i also showed a small, non-significant reduction in OxPL-PLG levels but not PLG, like high-intensity statin treatment and add-on ezetimibe. These findings suggest that lipid-lowering therapies may have a minor effect on OxPL modification of plasminogen, which could potentially enhance fibrinolysis and lower thrombotic risk, although this hypothesis requires direct functional studies. Overall, the clinical significance of changes in OxPL-PLG remains uncertain and warrants further investigation, especially given the central role of plasminogen in thrombosis and its emerging role as an OxPL carrier complementary to Lp(a).

### 4.4 Study limitations

Major limitations of this study include short follow-up, lack of randomization and absence of a placebo arm. The small sample size, especially in the PCSK9i group, and the inherent variability in Lp(a) and OxPL levels significantly limit the power and generalizability of this study as well as interpretation of non-significant findings. Therefore, these findings should be viewed as preliminary and hypothesis-generating and should be further validated with larger studies and longer follow-up periods. Furthermore, the lack of randomization and differences in baseline characteristics may have introduced confounding factors affecting treatment response despite statistical adjustment. Specifically, differences in baseline characteristics such as the higher prevalence of ASCVD and familial hypercholesterolemia in the PCSK9i group may have influenced OxPL dynamics independently of treatment effects. This non-randomized design limitation should be considered when interpreting study results. Patients who received add-on ezetimibe or add-on PCSK9i were already on a stable lipid-lowering regimen prior to the addition of these agents. Consequently, the results presented reflect the additive effect of ezetimibe or PCSK9i when incorporated into existing therapy, rather than their isolated efficacy. Finally, while we quantified OxPL enrichment per particle, we did not measure the total plasma OxPL burden, which could have provided further insight into the overall atherogenic and inflammatory potential.

## 5 Conclusions

Both high-intensity statins and add-on ezetimibe led to increases in OxPL-apoB and OxPL-apo(a) levels, while the addition of PCSK9i did not significantly affect these parameters during a 3-month follow-up. The clinical relevance of these findings should be explored in future studies with larger sample size and longer duration.

## Financial support

The present study was supported by a research grant from the 10.13039/501100010353Hellenic Atherosclerosis Society, which was received by Koutsogianni Amalia Despoina.

## Declaration of competing interest

Amalia Despoina Koutsogianni has received personal fees from Novartis, outside the submitted work. Fotios Barkas has received honoraria and personal fees from Amgen, Novartis, Novo Nordisk and Viatris, outside the submitted work. George Liamis has received honoraria, grants and nonfinancial support from Angelini, Bayer, Menarini, and Sanofi, outside the submitted work. Evangelos Liberopoulos reports personal fees and non-financial support from Amgen, personal fees from Servier, personal fees from Boehringer-Ingelheim, personal fees and non-financial support from AstraZeneca, personal fees from MSD, personal fees from Lilly, personal fees and non-financial support from Bayer, personal fees from Novartis, personal fees from Chiesi, outside the submitted work. Constantinos Tellis and Alexandros Tselepis report no conflicts of interest associated with the present work. Sotirios Tsimikas is a coinventor and receives royalties from patents owned by UCSD; is a cofounder and has an equity interest in Oxitope and its affiliates Kleanthi Diagnostics and Covicept Therapeutics; and has a dual appointment at UCSD, and Ionis Pharmaceuticals.
